# Two high-resolution structures of the human E3 ubiquitin ligase Siah1

**DOI:** 10.1107/S1744309113031448

**Published:** 2013-11-28

**Authors:** Vadim Rimsa, Thomas C. Eadsforth, William N. Hunter

**Affiliations:** a Division of Biological Chemistry and Drug Discovery, College of Life Sciences, University of Dundee, Dundee DD1 5EH, Scotland

**Keywords:** E3 ubiquitin ligase, seven-in-absentia homologue 1 (Siah1), zinc finger

## Abstract

Siah1 is an E3 ubiquitin ligase that contributes to proteasome-mediated degradation of multiple targets in key cellular processes and which shows promise as a therapeutic target in oncology. Structures of a truncated Siah1 bound to peptide-based inhibitors have been reported. Here, new crystallization conditions have allowed the determination of a construct encompassing dual zinc-finger subdomains and substrate-binding domains at significantly higher resolution. Although the crystals appear isomorphous, two structures present distinct states in which the spatial orientation of one zinc-finger subdomain differs with respect to the rest of the dimeric protein. Such a difference, which is indicative of conformational freedom, infers potential biological relevance related to recognition of binding partners. The crystallization conditions and improved models of Siah1 may aid future studies investigating Siah1–ligand complexes.

## Abstract 2

Two structures of human Siah1 at 1.95 and 1.58 Å resolution provide more complete models for this protein and identify conformational variability in the subdomain organization.

## 1. Introduction   

Post-translational modification by ubiquitination controls many cellular processes, including the regulation of proteasome-mediated protein degradation (Grabbe *et al.*, 2011 ▶; Krämer *et al.*, 2013 ▶). Ubiquitination requires a cascade of enzymes, with the target or substrate specificity finally defined by the E3 ubiquitin ligase component, a noncatalytic component of the active complex that is formed with an E2 ligase. Siah1 (seven-in-absentia homologue 1) is an E3 ligase belonging to the RING (really interesting new gene) domain family (Budhidarmo *et al.*, 2012 ▶). It acts as a scaffolding protein and interacts with a variety of different substrates for ubiquitination and subsequent degradation (Qi *et al.*, 2013 ▶). Other proteins regulate the activity of Siah1 and some assemble together with Siah1 into multi-component E3 ligase complexes. Siah1 functions in Ras, DNA-damage and hypoxia signalling pathways, and is implicated in various cancers (House *et al.*, 2009 ▶). Inhibition of Siah1 activity impairs tumour growth and metastasis, suggesting that the protein is a potential therapeutic target (Wong & Möller, 2013 ▶).

Siah1 consists of an N-terminal RING domain, two zinc-finger subdomains and a C-terminal substrate-binding domain (SBD; Polekhina *et al.*, 2002 ▶). The SBD is primarily responsible for dimer formation. A number of Siah1 structures without the RING domain have been determined, with a highest resolution of 2.2 Å (PDB entry 2a25; Santelli *et al.*, 2005 ▶). Other structures have lower resolutions of between 2.4 and 3.0 Å (PDB entries 1k2f, 2an6, 4i7b, 4i7c and 4i7d; Polekhina *et al.*, 2002 ▶; House *et al.*, 2006 ▶; Stebbins *et al.*, 2013 ▶).

In support of compound-screening studies and a structure-based approach to ligand development (Hunter, 2009 ▶), we sought crystals of Siah1 that would allow us to validate potential hits. Here, we present two new crystallization conditions for Siah1 which led to high-resolution structures. The resulting models contained a number of improvements, such as modelled flexible loop regions that were absent in earlier structures, and moreover despite being ‘isomorphous’ revealed structural heterogeneity in the orientation of a zinc-finger subdomain.

## 2. Methods   

### 2.1. Crystallization and data collection   

**Figure 1:**
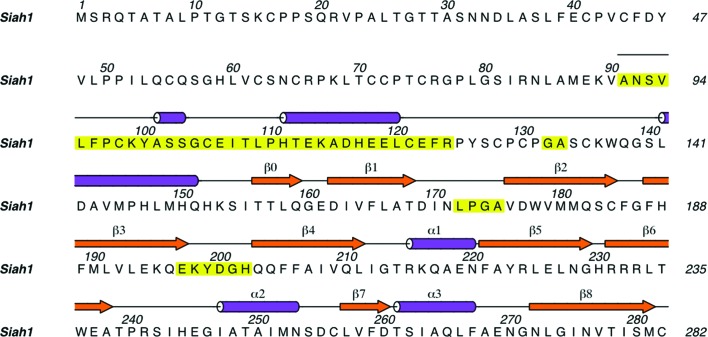
The primary and secondary structure of Siah1. The figure shows the full-length sequence of human Siah1 (UniProt entry Q8IUQ4). The secondary structure presented corresponds to residues 91–282 of the 1.95 Å resolution structure. The secondary-structure elements which are numbered make up the SBD. Residues that were modelled in the 1.95 Å resolution structure but that were absent in the search model used in molecular replacement are shown on a yellow background.

A gene fragment encoding human Siah1 without the RING domain (residues 91–282; Fig. 1 ▶; UniProt entry Q8IUQ4) was cloned and expressed and the product was purified as described previously (Rimsa *et al.*, 2013 ▶). The protein was screened for crystallization conditions using the high-throughput Phoenix liquid-handling system (Art Robbins Instruments/Rigaku) with commercially available formulations. Crystallization occurred at room temperature within 3 d in several conditions. Two of these conditions, No. 62 from The Classics Suite (Qiagen) and No. 86 from the Helsinki Random Screen 1 (Biocenter Finland), were refined using a hanging-drop vapour-diffusion method to yield diffraction-quality crystals. Optimized crystals grew from equal volumes of protein solution (15 mg ml^−1^ in 15 m*M* Tris–HCl pH 7.5, 30 m*M* NaCl, 10 m*M* DTT) and two reservoirs. The first condition optimized to a reservoir (reservoir I) consisting of 100 m*M* MES pH 6.5, 1.5 *M* MgSO_4_, while crystals were subsequently obtained using reservoir II (100 m*M* HEPES pH 7.0, 1.45 *M* Li_2_SO_4_). The crystals exhibited similar shapes (multi-faced prisms) and sizes (300 × 150 × 150 µm) irrespective of the reservoir used. The crystals were cryoprotected by the addition of 20%(*v*/*v*) glycerol to the appropriate reservoir prior to flash-cooling in liquid nitrogen and data collection at approximately 100 K. The diffraction properties of the first crystals obtained were characterized in-house and a data set was collected using a Rigaku MicroMax-007 copper-anode X-ray generator coupled to an R-AXIS IV^++^ dual image-plate detector. Subsequently, after the second crystallization condition had been identified a data set was collected using synchrotron radiation on beamline I03 at Diamond Light Source with a Pilatus 6M-F detector.

### 2.2. Structure solution and refinement   

Diffraction data sets were indexed and integrated with *iMosflm* (Battye *et al.*, 2011 ▶) and scaled using *SCALA* (Evans, 2006 ▶). The unit-cell parameters and symmetry suggested that the asymmetric unit consisted of a dimer with a *V*_M_ of 2.99 Å^3^ Da^−1^ and about 60% bulk solvent. Structures were determined by molecular replacement using *Phaser* (McCoy *et al.*, 2007 ▶). The first structure, determined to 1.95 Å resolution, used the coordinates of a Siah1 monomer (PDB entry 2a25, chain *A*; Santelli *et al.*, 2005 ▶) as a search model. The output model from molecular replacement identified the dimer previously observed and was subjected to a single cycle of rigid-body and restrained refinement using *REFMAC*5 (Murshudov *et al.*, 2011 ▶). All atoms were assigned a starting *B* factor derived from the Wilson plot statistics generated in *SCALA*. Electron-density and difference density maps were inspected and model extension and fitting were carried out in *Coot* (Emsley *et al.*, 2010 ▶). This was followed by further cycles of refinement and model fitting, which resulted in the inclusion of 30–35 residues at the N-terminal end and three internal regions which were absent from the search model (Fig. 1 ▶). Zinc ions, water molecules, other ligands and side-chain rotamers were included once the protein model had been completed. The presence of the Zn^2+^ ions was suggested by the chemical environment, the electron density and from previous studies of zinc-finger proteins. We confirmed the assignment with a single-wavelength anomalous dispersion analysis using diffraction data recorded at the X-ray absorption edge of zinc (data not shown). The source of the zinc ions was presumed to be the broth used to culture the *E. coli* cells. Tight local noncrystallographic symmetry restraints were used in the early rounds of refinement, which were subsequently released. TLS (translation/libration/screw) parameters obtained from the *TLSMD* server (Painter & Merritt, 2006 ▶) were applied in the later stages of refinement. The coordinates of this structure were subsequently used to solve the second structure of Siah1 to 1.58 Å resolution. The *B* factors were reset to the Wilson *B* value and refinement was carried out as described above but, with higher resolution data available, anisotropic temperature factors were refined. It was noted that significant changes to the model were required in the region of one N-terminal zinc-finger domain. Model geometry was monitored during refinement with *MolProbity* (Chen *et al.*, 2010 ▶). Structure superpositions were calculated using *Secondary Structure Matching* (Krissinel & Henrick, 2007 ▶) in *Coot* or *LSQKAB* (Kabsch, 1976 ▶), and the *Protein Interfaces, Surfaces and Assemblies* (*PISA*) server (Krissinel & Henrick, 2007 ▶) was used to characterize the dimer interface. Secondary structure was assigned using *Coot* and by inspection. Figures were prepared using *PyMOL* (DeLano, 2002 ▶) and *ALINE* (Bond & Schüttelkopf, 2009 ▶). Data-collection and refinement statistics are shown in Table 1 ▶.

## 3. Results and discussion   

### 3.1. Quality of the structures   

Two isomorphous crystal structures of Siah1 were determined at 1.95 and 1.58 Å resolution. There are two molecules per asymmetric unit, labelled chains *A* and *B*, and they form a dimer consistent both with the observation that a single species of approximate mass 39 kDa was noted during size-exclusion chromatography purification and with published structures of Siah1 (House *et al.*, 2003 ▶; Polekhina *et al.*, 2002 ▶; Santelli *et al.*, 2005 ▶; Stebbins *et al.*, 2013 ▶). Continuous well defined electron density is observed for the polypeptide backbone in the 1.95 Å resolution structure, corresponding to residues 91–282 of the native Siah1 sequence. Here, chain *A* also contains an additional methionine at the N-terminus, which is a cloning artifact. The electron-density map was very well defined in the 1.58 Å resolution structure, apart from one loop region (Tyr199–Gly201 in chain *A* and Lys198–Tyr199 in chain *B*), and these residues were excluded from the model. A Ramachandran plot shows that 97.4% of the residues in the high-resolution structure are located in the most favoured region, with no outliers. One outlier is present in the lower resolution structure and 96.9% of the amino acids are situated in the most favoured region. The diffraction-component precision indicator (DPI; Cruickshank, 1999 ▶) values (Table 1 ▶) are 0.073 and 0.149 Å for the 1.58 and 1.95 Å resolution structures, respectively. The DPI values for PDB entries 2a25, 4i7c, 4i7d and 1k2f, as determined by the Electron Density Server (Kleywegt *et al.*, 2004 ▶), are 0.233, 0.470, 0.234 and 0.186 Å, respectively.

### 3.2. Overall structure and comparisons with previous Siah1 models   

**Figure 2:**
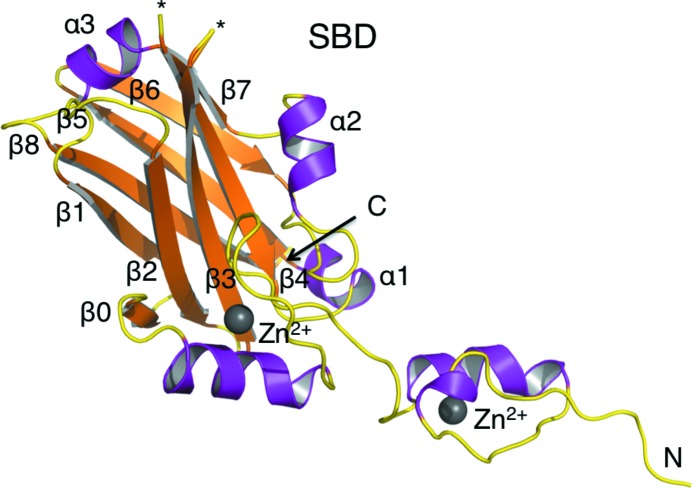
Secondary-structure elements of the Siah1 monomer. Ribbon diagram showing the SBD and two zinc-finger subdomains of the 1.58 Å resolution structure. The α-­helices and β-strands are coloured purple and orange, respectively. The N- and C-­terminal ends are labelled, as are the secondary-structure elements. Asterisks mark the positions of disordered loops.

**Figure 3:**
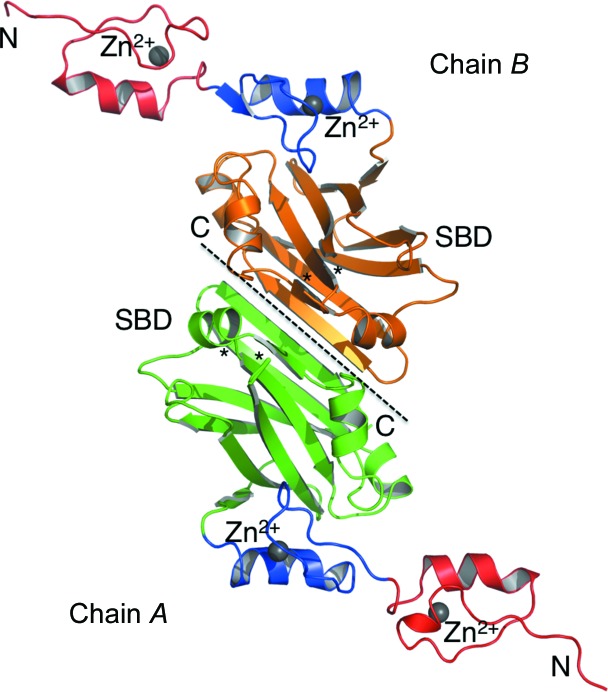
Overall structure of the Siah1 dimer. The figure shows the 1.58 Å resolution structure. The zinc-finger subdomains are coloured red and blue, while the substrate-binding domains (SBDs) of monomers *A* and *B* are coloured green and orange, respectively. The N- and C-termini are labelled. The Zn^2+^ ions are shown as grey spheres and the disordered loops are marked with asterisks.

Each Siah1 subunit consists of two zinc-finger subdomains and a C-­terminal substrate-binding domain (SBD; Fig. 2 ▶). The N-terminal zinc-finger motif belongs to the Cys_2_HisCys class of zinc fingers, while the second constitutes a member of the Cys_2_His_2_ type. The SBD forms a dimerization interface with its counterpart from the partner subunit to create a Z-shaped structure (Fig. 3 ▶). The SBD consists of two antiparallel β-sheets of four strands each placed on top of one another. We use the established numbering scheme from previous work (Polekhina *et al.*, 2002 ▶). The first β-sheet consists of strands β2, β3, β4 and β7, while the second consists of strands β1, β8, β5 and β6. An additional strand β0 is parallel to β2, connecting the SBD to an adjacent zinc finger. Furthermore, the SBD contains three short α-­helices (α1–α3) that link β4 to β5, β6 to β7, and β7 to β8.

**Figure 4:**
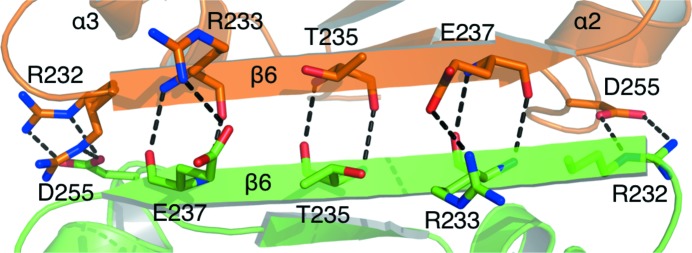
The dimer interface, depicting some of the residues forming the intermolecular hydrogen bonding. Monomers are coloured green and orange. Nitrogen and oxygen positions are coloured blue and red, respectively.

The total surface area of a monomer is about 10 800 Å^2^. The buried area between the two subunits is 1100 Å^2^; therefore, approximately 10% of the surface area is involved in dimerization. Siah1 dimerization involves the C-terminal regions of the SBD and about a dozen hydrogen bonds and salt bridges. The majority of these intermolecular contacts occur between residues on β6 of each subunit. For example, Arg232 and Arg233 of one monomer establish salt-bridge interactions with Asp255 and Glu237 of the partner subunit (Fig. 4 ▶). The equivalent arginine residues in the partner subunit make the same contacts with the residues in the first subunit. A further six hydrogen bonds are formed between the main chains of these two antiparallel β6 strands, involving Arg233, Thr235 and Glu237. In addition, the subunit–subunit interactions are mediated by a number of water molecules (data not shown). The extensive network of intermolecular contacts and the large buried interface suggest a tight association of the two monomers.

**Figure 5:**
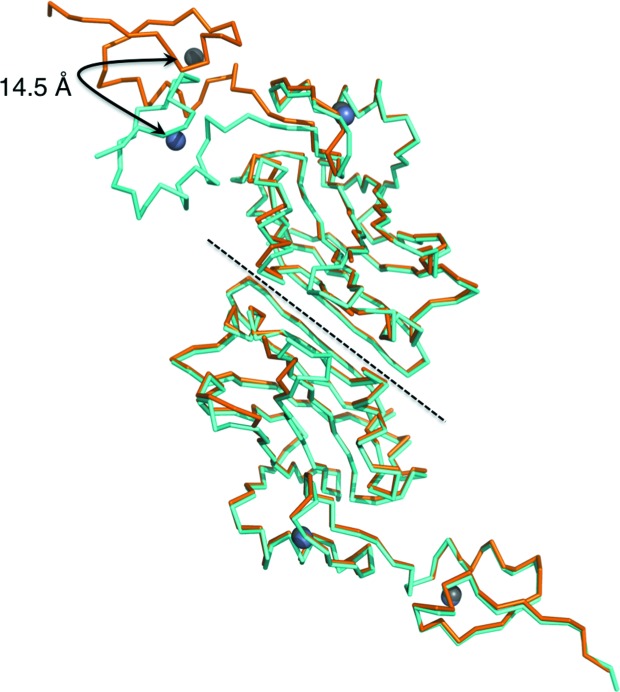
Overlay of the two Siah1 structures. The 1.95 and 1.58 Å resolution structures are shown in cyan and orange, respectively, with their associated Zn^2+^ ions as grey spheres. The Zn^2+^ ion in the N-terminal zinc-finger motif is displaced by 14.5 Å between the two structures.

**Figure 6:**
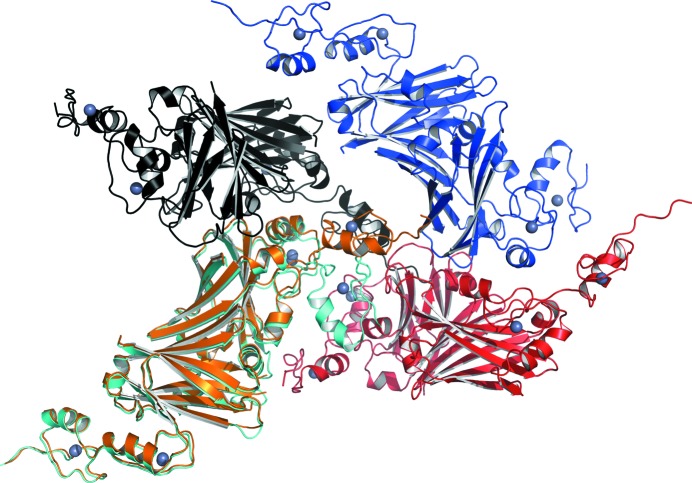
The crystal-packing environment of the N-terminal zinc finger of the *B* chain of Siah1. The overlaid 1.95 and 1.58 Å resolution structures are coloured cyan and orange, respectively. Three symmetry-related molecules of the 1.95 Å resolution structure are coloured red (1 − *x*, 1 − *y*, *z*), blue (1 − *x* + ½, *y* + ½, −*z* + ½) and black (*x* + ½, −*y* + ½, −*z* + ½). The Zn^2+^ ions are shown as grey spheres.

The degree of NCS is more highly conserved in the high-resolution structure, in which the superposition of chain *A* on chain *B* (171 C^α^ positions) results in an r.m.s.d. of 1.36 Å. In the lower resolution structure the r.m.s.d. is 5.36 Å (181 C^α^ positions). We became aware of significant differences at an early stage of refinement and this indicated that the NCS restraints should be released. Inspection confirmed that the NCS breakdown is owing to differences in the orientation of the N-terminal zinc-finger subdomain with respect to the rest of the protein. Superposition of the two new structures on each other gives r.m.s.d. values of 0.80 and 4.89 Å for chains *A* (175 C^α^ atoms) and *B* (164 C^α^ atoms), respectively. The high value again occurs owing to a difference in the position of the N-terminal zinc-finger subdomain in the two structures (Fig. 5 ▶). The result is that the Zn^2+^ ions are displaced by about 14.5 Å when the structures are superposed. The overall molecular packing in each lattice remains well conserved and it is simply that there is space that allows the N-­terminal zinc finger of the *B* chain to adopt a position where it can either interact with a molecule at 1 − *x* + ½, *y* + ½, −*z* + ½ as in the highest resolution structure or interact with a molecule at 1 − *x*, 1 − *y*, *z* as in the case of the lower resolution structure (Fig. 6 ▶). The space vacated is then occupied by bulk solvent.

Superpositions with previously determined structures in the PDB identify the same feature. Superposition of Siah1 chains excluding the N-terminal zinc fingers give lower r.m.s.d. values, typically in the range 0.5–1.0 Å for about 135 C^α^ atoms; for the few structures where the zinc-finger subdomain is included the r.m.s.d. values increase to between 2.6 and 4.8 Å. The notable conformational flexibility of the N-terminal zinc-finger subdomain may contribute to its role in the biological function by adapting its position to assist in interactions with distinct substrate proteins. Further work would be required to address such a hypothesis.

### 3.3. Siah1 interactions with other proteins and mode of inhibition   

Siah1 interacts with a variety of protein targets as well as the binding partners that control the contributions to ubiquitination. For example, Siah1 alone is able to polyubiquitinate β-catenin, a key component of the Wnt [the name is a combination of the homologous genes Wg (wingless) and Int] signalling pathway controlling cell fate and proliferation (Dimitrova *et al.*, 2010 ▶). However, this process is much more efficient when Siah1 forms an E3 ligase complex with SIP (Siah-interacting protein), the adaptor protein Skp1 and the F-box protein Ebi (Matsuzawa & Reed, 2001 ▶). Many Siah1-interacting proteins carry a conserved binding motif V*x*P, where *x* is any residue (House *et al.*, 2003 ▶). The motif is present in SIP and its crucial role in facilitating interaction of SIP with Siah1 has been shown in a number of biophysical studies (Santelli *et al.*, 2005 ▶; Bhattacharya *et al.*, 2005 ▶). As an aside, we note that full-length human SIP was expressed and purified for structural studies in complex with Siah1. The interaction between the two proteins, with one-to-one stoichiometry (one Siah1 dimer and two SIP molecules), was confirmed by size-exclusion gel chromatography and the complex was purified, but crystallization experiments have so far failed to yield any positive results.

The currently available inhibitors of Siah1 are short peptides that contain the V*x*P motif in their sequences (House *et al.*, 2003 ▶; Stebbins *et al.*, 2013 ▶). These peptides have been shown to reduce tumour growth and metastasis in a number of model systems (Möller *et al.*, 2009 ▶; Qi *et al.*, 2010 ▶). Furthermore, they provide good starting points for the design of novel Siah1 inhibitors, and a number of covalent peptide-based inhibitors with improved affinities have recently been reported (Stebbins *et al.*, 2013 ▶). Access to crystallization conditions that lead to high-resolution diffraction data as reported here might in future assist in the structural characterization of novel inhibitors in support of early-stage drug discovery.

## Supplementary Material

PDB reference: Siah1, 4c9z

PDB reference: 4ca1

## Figures and Tables

**Table 1:** Crystallographic statistics Values in parentheses are for the highest resolution shell.

PDB code	4c9z	4ca1
Space group	*I*222	*I*222
Unit-cell parameters ()	*a* = 75.14, *b* = 104.59, *c* = 133.16	*a* = 76.06, *b* = 104.12, *c* = 133.59
Resolution ()	44.991.95 (2.061.95)	52.061.58 (1.671.58)
No. of reflections recorded	141688 (11394)	359712 (50840)
Unique reflections	38340 (5357)	72738 (10523)
Completeness (%)	99.3 (96.0)	100.0 (100.0)
Multiplicity	3.7 (2.1)	4.9 (4.8)
*I*/(*I*)	19.3 (4.5)	16.1 (3.5)
Mosaicity ()	0.4	0.5
Wilson *B* factor (^2^)	29.2	19.1
Rotation per frame ()	0.5	0.2
Total rotation range ()	100	148
Exposure per frame (s)	600	0.2
Crystal-to-detector distance (mm)	160.0	268.8
Wavelength ()	1.5418	0.9763
Residues		
Chain *A*	91282	91198, 202282
Chain *B*	95282	91197, 200282
No. of water molecules	218	331
No. of Tris molecules	2	0
No. of glycerol molecules	3	12
No. of sulfate ions	3	5
No. of Cl ions	4	3
No. of Zn^2+^ ions	4	4
*R*_merge_ † (%)	3.7 (17.8)	5.2 (47.8)
*R*_work_ ‡ (%)	19.8	14.1
*R*_free_ § (%)	23.9	18.9
Average *B* factor for all atoms ()	18.7	22.9
Cruickshank DPI¶ ()	0.149	0.073
Ramachandran plot††		
Most favoured (%)	96.9	97.4
Additional allowed (%)	2.8	2.6
Outliers (%)	0.3	0.0
R.m.s.d. on ideal values‡‡		
Bond length ()	0.01	0.02
Bond angle ()	1.32	2.34

† *R*_merge_ = 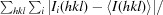 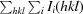, where *I_i_*(*hkl*) is the intensity of the *i*th measurement of reflection *hkl* and *I*(*hkl*) is the mean value of *I_i_*(*hkl*) for all *i* measurements.

‡ *R*_work_ = 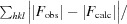 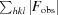, where *F*_obs_ is the observed structure-factor amplitude and *F*_calc_ is the structure-factor amplitude calculated from the model.

§ *R*_free_ is the same as *R*_work_ except calculated with a subset (5%) of data that were excluded from refinement calculations.

¶ Diffraction-component precision indicator (Cruickshank, 1999 ▶).

†† Chen *et al.* (2010 ▶).

‡‡ Engh Huber (1991 ▶).

## References

[bb1] Battye, T. G. G., Kontogiannis, L., Johnson, O., Powell, H. R. & Leslie, A. G. W. (2011). *Acta Cryst.* D**67**, 271–281. 10.1107/S0907444910048675 PMC3069742 21460445

[bb2] Bhattacharya, S., Lee, Y.-T., Michowski, W., Jastrzebska, B., Filipek, A., Kuznicki, J. & Chazin, W. J. (2005). *Biochemistry*, **44**, 9462–9471. 10.1021/bi0502689 15996101

[bb3] Bond, C. S. & Schüttelkopf, A. W. (2009). *Acta Cryst.* D**65**, 510–512. 10.1107/S0907444909007835 19390156

[bb4] Budhidarmo, R., Nakatani, Y. & Day, C. L. (2012). *Trends Biochem. Sci.* **37**, 58–65. 10.1016/j.tibs.2011.11.001 22154517

[bb5] Chen, V. B., Arendall, W. B., Headd, J. J., Keedy, D. A., Immormino, R. M., Kapral, G. J., Murray, L. W., Richardson, J. S. & Richardson, D. C. (2010). *Acta Cryst.* D**66**, 12–21. 10.1107/S0907444909042073 PMC2803126 20057044

[bb6] Cruickshank, D. W. J. (1999). *Acta Cryst.* D**55**, 583–601. 10.1107/s0907444998012645 10089455

[bb7] DeLano, W. L. (2002). *PyMOL* http://www.pymol.org.

[bb8] Dimitrova, Y. N., Li, J., Lee, Y.-T., Rios-Esteves, J., Friedman, D. B., Choi, H.-J., Weis, W. I., Wang, C.-Y. & Chazin, W. J. (2010). *J. Biol. Chem.* **285**, 13507–13516. 10.1074/jbc.M109.049411 PMC2859511 20181957

[bb9] Emsley, P., Lohkamp, B., Scott, W. G. & Cowtan, K. (2010). *Acta Cryst.* D**66**, 486–501. 10.1107/S0907444910007493 PMC2852313 20383002

[bb10] Engh, R. A. & Huber, R. (1991). *Acta Cryst.* A**47**, 392–400.

[bb11] Evans, P. (2006). *Acta Cryst.* D**62**, 72–82. 10.1107/S0907444905036693 16369096

[bb12] Grabbe, C., Husnjak, K. & Dikic, I. (2011). *Nature Rev. Mol. Cell Biol.* **12**, 295–307. 10.1038/nrm3099 PMC3654194 21448225

[bb13] House, C. M., Frew, I. J., Huang, H.-L., Wiche, G., Traficante, N., Nice, E., Catimel, B. & Bowtell, D. D. L. (2003). *Proc. Natl Acad. Sci. USA*, **100**, 3101–3106. 10.1073/pnas.0534783100 PMC152253 12626763

[bb32] House, C. M., Hancock, N. C., Moller, A., Cromer, B. A., Fedorov, V., Bowtell, D. D. L., Parker, M. W. & Polekhina, G. (2006). *Structure*, **14**, 695–701. 10.1016/j.str.2005.12.013 16615911

[bb14] House, C. M., Möller, A. & Bowtell, D. D. (2009). *Cancer Res.* **69**, 8835–8838. 10.1158/0008-5472.CAN-09-1676 19920190

[bb15] Hunter, W. N. (2009). *J. Biol. Chem.* **284**, 11749–11753. 10.1074/jbc.R800072200 PMC2673241 19103598

[bb16] Kabsch, W. (1976). *Acta Cryst.* A**32**, 922–923.

[bb17] Kleywegt, G. J., Harris, M. R., Zou, J., Taylor, T. C., Wählby, A. & Jones, T. A. (2004). *Acta Cryst.* D**60**, 2240–2249. 10.1107/S0907444904013253 15572777

[bb18] Krämer, O. H., Stauber, R. H., Bug, G., Hartkamp, J. & Knauer, S. K. (2013). *Leukemia*, **27**, 792–802. 10.1038/leu.2012.284 23038274

[bb19] Krissinel, E. & Henrick, K. (2007). *J. Mol. Biol.* **372**, 774–797. 10.1016/j.jmb.2007.05.022 17681537

[bb20] Matsuzawa, S. I. & Reed, J. C. (2001). *Mol. Cell*, **7**, 915–926. 10.1016/s1097-2765(01)00242-8 11389839

[bb21] McCoy, A. J., Grosse-Kunstleve, R. W., Adams, P. D., Winn, M. D., Storoni, L. C. & Read, R. J. (2007). *J. Appl. Cryst.* **40**, 658–674. 10.1107/S0021889807021206 PMC2483472 19461840

[bb22] Möller, A., House, C. M., Wong, C. S. F., Scanlon, D. B., Liu, M. C. P., Ronai, Z. & Bowtell, D. D. L. (2009). *Oncogene*, **28**, 289–296. 10.1038/onc.2008.382 PMC3000903 18850011

[bb23] Murshudov, G. N., Skubák, P., Lebedev, A. A., Pannu, N. S., Steiner, R. A., Nicholls, R. A., Winn, M. D., Long, F. & Vagin, A. A. (2011). *Acta Cryst.* D**67**, 355–367. 10.1107/S0907444911001314 PMC3069751 21460454

[bb24] Painter, J. & Merritt, E. A. (2006). *J. Appl. Cryst.* **39**, 109–111.

[bb25] Polekhina, G., House, C. M., Traficante, N., Mackay, J. P., Relaix, F., Sassoon, D. A., Parker, M. W. & Bowtell, D. D. L. (2002). *Nature Struct. Biol.* **9**, 68–75. 10.1038/nsb743 11742346

[bb26] Qi, J., Kim, H., Scortegagna, M. & Ronai, Z. A. (2013). *Cell Biochem. Biophys.* **67**, 15–24. 10.1007/s12013-013-9636-2 PMC3758783 23700162

[bb27] Qi, J., Pellecchia, M. & Ronai, Z. A. (2010). *Oncotarget*, **1**, 379–385. 10.18632/oncotarget.171 PMC2964873 21037926

[bb28] Rimsa, V., Eadsforth, T. C. & Hunter, W. N. (2013). *PLoS One*, **8**, e69538. 10.1371/journal.pone.0069538 PMC3720700 23936039

[bb29] Santelli, E., Leone, M., Li, C., Fukushima, T., Preece, N. E., Olson, A. J., Ely, K. R., Reed, J. C., Pellecchia, M., Liddington, R. C. & Matsuzawa, S. (2005). *J. Biol. Chem.* **280**, 34278–34287. 10.1074/jbc.M506707200 16085652

[bb30] Stebbins, J. L., Santelli, E., Feng, Y., De, S. K., Purves, A., Motamedchaboki, K., Wu, B., Ronai, Z. A., Liddington, R. C. & Pellecchia, M. (2013). *Chem. Biol.* **20**, 973–982. 10.1016/j.chembiol.2013.06.008 PMC3763817 23891150

[bb31] Wong, C. S. F. & Möller, A. (2013). *Cancer Res.* **73**, 2400–2406. 10.1158/0008-5472.CAN-12-4348 23455005

